# Homo-Tris-Nitrones Derived from α-Phenyl-*N-tert*-butylnitrone: Synthesis, Neuroprotection and Antioxidant Properties

**DOI:** 10.3390/ijms21217949

**Published:** 2020-10-26

**Authors:** Daniel Diez-Iriepa, Beatriz Chamorro, Marta Talaván, Mourad Chioua, Isabel Iriepa, Dimitra Hadjipavlou-Litina, Francisco López-Muñoz, José Marco-Contelles, María Jesús Oset-Gasque

**Affiliations:** 1 Laboratory of Medicinal Chemistry, Institute of Organic Chemistry (CSIC), Juan de la Cierva 3, 28006 Madrid, Spain; daniel.diezi@uah.es (D.D.-I.); mchioua@gmail.com (M.C.); 2 Department of Organic Chemistry and Inorganic Chemistry, Alcalá University, Alcalá de Henares, 28805 Madrid, Spain; isabel.iriepa@uah.es; 3 Department of Biochemistry and Molecular Biology, Faculty of Pharmacy, Complutense University of Madrid, Plaza Ramón y Cajal s/n, Ciudad Universitaria, 28040 Madrid, Spain; beatrcha@ucm.es (B.C.); martalav@ucm.es (M.T.); 4 Faculty of Health, Camilo José Cela University of Madrid (UCJC), 28692 Villafranca del Castillo, Spain; flopez@ucjc.edu; 5 Institute of Chemical Research Andrés M. del Río, Alcalá University, Alcalá de Henares, 28805 Madrid, Spain; 6 Department of Pharmaceutical Chemistry, School of Pharmacy, Faculty of Health Sciences, Aristotle University of Thessaloniki, 54124 Thessaloniki, Greece; hadjipav@pharm.auth.gr; 7 Neuropsychopharmacology Unit, “Hospital 12 de Octubre” Research Institute, 28041 Madrid, Spain; 8 Instituto Universitario de Investigación en Neuroquímica, Universidad Complutense de Madrid, 28040 Madrid, Spain

**Keywords:** antioxidants, free radical scavengers, homo-tris-nitrones, neuroprotection, nitrones, oligomycin A/rotenone, oxygen-glucose-deprivation model, α-phenyl-*N-tert*-butylnitrone, synthesis

## Abstract

Herein we report the synthesis, antioxidant and neuroprotective power of homo-tris-nitrones (**HTN**) **1-3**, designed on the hypothesis that the incorporation of a third nitrone motif into our previously identified homo-bis-nitrone **6** (**HBN6**) would result in an improved and stronger neuroprotection. The neuroprotection of **HTNs 1-3**, measured against oligomycin A/rotenone, showed that **HTN2** was the best neuroprotective agent at a lower dose (EC_50_ = 51.63 ± 4.32 μM), being similar in EC_50_ and maximal activity to α-phenyl-*N*-*tert*-butylnitrone (**PBN**) and less potent than any of **HBNs 4-6**. The results of neuroprotection in an in vitro oxygen glucose deprivation model showed that **HTN2** was the most powerful (EC_50_ = 87.57 ± 3.87 μM), at lower dose, but 50-fold higher than its analogous **HBN5**, and ≈1.7-fold less potent than **PBN**. **HTN3** had a very good antinecrotic (IC_50_ = 3.47 ± 0.57 μM), antiapoptotic, and antioxidant (EC_50_ = 6.77 ± 1.35 μM) profile, very similar to that of its analogous **HBN6**. In spite of these results, and still being attractive neuroprotective agents, **HTNs 2** and **3** do not have better neuroprotective properties than **HBN6**, but clearly exceed that of **PBN**.

## 1. Introduction

Nowadays it is largely accepted that reactive oxygen species (ROS), such as O_2_^−•^, HO^•^, HO_2_^•^, are one of the main biological factors involved in the etiology of a number of pathologies such as neurodegenerative and psychiatric disorders, or stroke, playing key roles in their pathogenesis, suggesting thus possible therapeutic interventions [1,2,3]. Consequently, the identification of new antioxidants, and the development of more potent analogues are under extensive research [4].

Stroke is a major public health problem, whose current clinical treatment, based on treatment with recombinant tissue plasminogen activator is hampered by important side effects and narrow therapeutic window [5]. Thus, there is an urgent need for more effective therapies. This is the reason, combined to the multifactorial nature of this pathology, of the increased and continued attention that has been devoted to the research for new and more effective ROS trapping agents [6].

**Figure 1:**
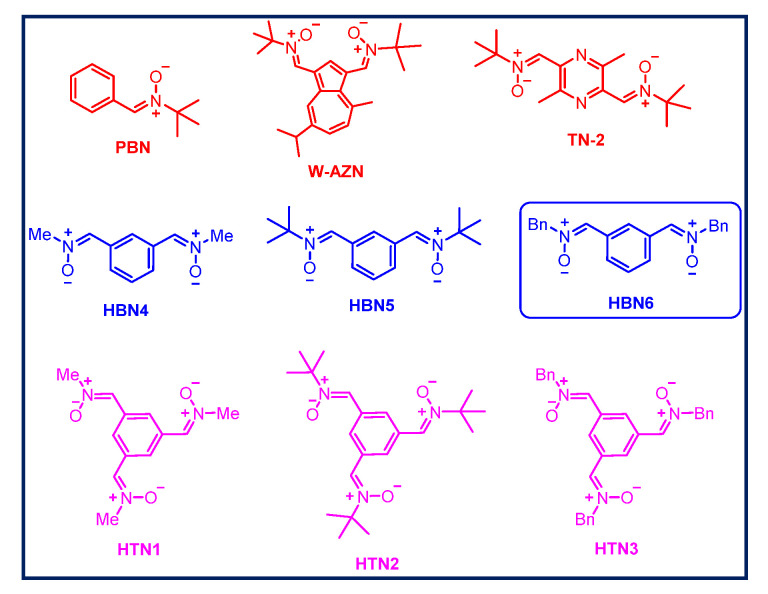
Structures of α-phenyl-*N*-*tert*-butylnitrone (PBN), W-AZN, TN-2 (red), homo-bis-nitrones (HBNs 4-6) (blue), and homo-tris-nitrones (HTNs 1-3) (pink).

Nitrones, well known antioxidant organic molecules widely used as radical traps, appear to be interesting drug candidates to treat stroke [7,8,9,10,11,12,13,14,15,16,17,18,19]. The first proposal of nitrones as therapeutic candidates for stroke was made by Novelli, who reported that α-phenyl *N*-*tert*-butylnitrone (**PBN**) (Figure 1) prevented and reversed traumatic shock injury in rats [20]. Thus, not surprisingly, the search for new nitrones continue to attract the interest of a number of laboratories around the world [21,22,23,24,25,26,27,28,29,30,31,32,33]. This has been the case of our group, that in the last decade has been very active in this area reporting new antioxidant nitrones for the potential stroke therapy [34,35,36,37,38].

In this field, the neuroprotective profile and strong antioxidant capacity of bis-nitrones, as well as their potential clinical applications for the therapy of stroke has been documented in the literature, showing very promising hit-azulenyl nitrones [39,40,41] such as bis-nitrone **W-AZN** (Figure 1), of particular interest due to its neuroprotective power in an in vivo model of stroke [42], or bis-nitrone **TN-2** (Figure 1), an excellent example of a compound bearing two *tert*-butyl nitrone groups, bound to a pyrazine heterocyclic nucleus, with remarkable neuroprotective capacity most possibly due to its ability to trap toxic ROS [43,44,45].

Our group has recently described a number of homo-bis-nitrones (**HBNs**) derived from **PBN**, from which **HBN6** (Figure 1) exhibited a potent neuroprotective effect in in vitro and in vivo ischemia models, a very good antinecrotic and antiapoptotic activity, as well as very good antioxidant properties, stronger than the parent **PBN [46]**. Thus, our previous results strongly suggest that **HBN6** is a potential neuroprotective agent against stroke and that the introduction of two nitrone groups improves the neuroprotective properties of **PBN**-based nitrone containing only one nitrone group [46]. Now, and continuing with our interest of polynitrones for stroke, and based on these previously reported results [46], we wondered whether the incorporation of a third nitrone motif would result in an improved and stronger neuroprotection effect. Consequently, we designed symmetrically substituted homo-tris-nitrones (**HTNs**) resulting in nitrones **HTNs 1**-**3** (Figure 1).

## 2. Results and Discussion

### 2.1. Chemistry

**Scheme 1:**
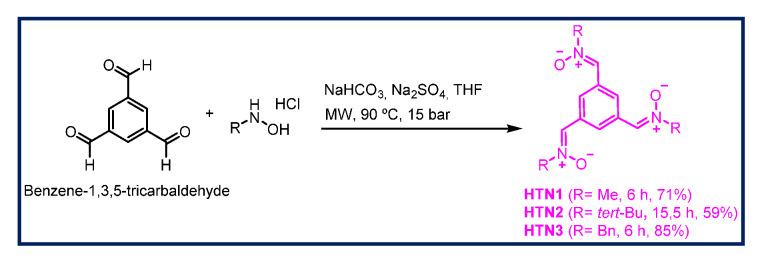
Synthesis of HTNs 1-3.

The synthesis of **HTNs 1**-**3** was carried out as shown in Scheme 1 starting form benzene-1,3,5-tricarbaldehyde, and readily available *N*-alkylhydroxylamine hydrochlorides were selected. All the nitrones were isolated as pure and single *Z* isomers at the double ArC(H)=N^+^(O^-^)R bond, and gave analytical and spectroscopic data in good agreement with their structures (see Materials and Methods, Supplementary Material). Only **HTN2** (Figure 1) has been previously described [47,48] in studies connected with the synthesis and magnetic properties of stable nitroxyl tri-radicals [47], and as spin trapping agents for the treatment of diseases associated with oxidation of lipids and proteins [48]. According to our design, and taking into account the structure if **HBN6** as reference, **HTNs 1**-**3** all bear the nitrone motives in relative *meta* position, a choice that was favored by the readily, commercially available benzene-1,3,5-tricarbaldehyde precursor, **HTN1**, **HTN2,** and **HTN3** (Figure 1) bearing a *N*-methyl, a *N*-*tert*-butyl, and a *N*-benzyl group, respectively, at the R nitrone functional group.

### 2.2. Neuroprotection Studies of HTNs 1-3

#### 2.2.1. Neuroprotection Analysis in an Oligomycin A/Rotenone Model

One of the first events that take place in the initial stages of stroke is the collapse of the mitochondrial electron transport chain, leading to extended cell death and brain damage, due to the formation of ROS. In order to mimic this event into suitable experiments, we tested the effect of the new nitrones on cell death induced by the neurotoxic combination of oligomycin A/rotenone (O/R), inhibitors of mitochondrial complexes V and I, respectively. Based on a previous work from our laboratory [46], we selected the appropriate experimental conditions, and tested the neuroprotective effect of **HTNs 1-3** at different concentrations (1–1000 μM), adding them 10 min before the administration of O10 μM/R30 μM, and using **PBN** and N-acetyl-*L*-cysteine (NAC) as reference compounds [46].

With pure nitrones **HTNs 1**-**3** in hand, we next investigated in depth their neuroprotective capacity according to the usual experimental protocols (see Materials and Methods) [49,50,51,52].

**Figure 2:**
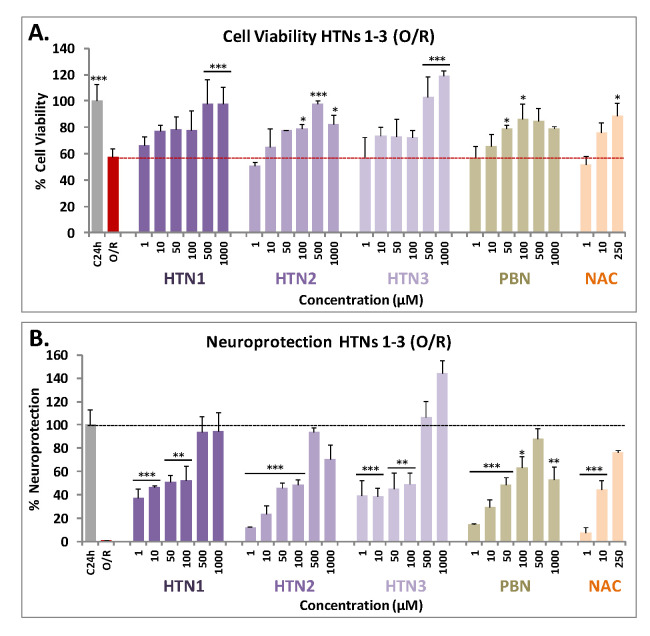
Neuroprotective effect of HTNs 1-3 on SH-SY5Y human neuroblastoma metabolic activity after treatment with oligomycin A 10 μM/rotenone 30 μM (O/R). (**A**) Bars show % cell viability after treatment with O/R, with, or without, HBNs, PBN, and NAC, at the indicated concentrations. (**B**) Bars show % of the resulting neuroprotection at the same concentrations, considering as 100% neuroprotection the difference between the control (C24h) viability and the obtained in the presence of O/R. Values are the mean ± SEM of three experiments, each one performed in triplicate. The statistics in (**A**) compare differences with O/R (red dotted line) and the statistics in (**B**) compare differences with control (100% neuroprotection without nitrones) (C24h) (black dotted line) at * *p* < 0.05, ** *p* < 0.01, and *** *p* < 0.001 (one-way ANOVA, followed by Holm−Sidak analysis as a test post hoc). No statistic comparisons are shown for values higher than those for C.

As shown in Figure 2A, **HTNs 1**-**3** showed a clear effect increasing cell viability in a concentration-dependent manner. The nitrone that provided the best result was **HTN3**, since from very low concentrations (10 μM) achieves a viability value above 70%, and even at high concentrations exceeds the viability of the control. **HTN1,** at low concentrations (1 μM), provided more than 60% viability, although like **HTN2**, failed to reach the maximal cell viability observed for **HTN3** at high concentrations. In general, among the **HTNs** we could not observe high significant differences, but all of them improve the data obtained for the reference nitrone, **PBN**. Regarding neuroprotection, it was observed that the neuroprotective effects of **HTNs** were clearly concentration dependent (Figure 2B).

To assess more accurately the differences in neuroprotective capacity of these nitrones, a study of the EC_50_ was carried out (Table 1). The order of neuroprotective potency of the compounds (from lowest to highest EC_50_) was **HTN2** ≥ **PBN** ≥ **HTN3** ≥ **HTN1** (Table 1). However, the maximal activity (%) data reflect a different trend (from highest to lowest): **HTN3** ≥ **HTN1** ≥ **PBN** ≥ **HTN2**. This means that the best compound that produces neuroprotection at a lower dose is **HTN2**, bearing a *N*-*tert*-Bu substituent; while the compound that produces a greater neuroprotection, although at a higher dose, is **HTN3**, bearing a *N*-Bn substituent. **HTN1**, bearing a *N*-Me substituent, would have an intermediate capacity between its analogues **HTN2** and **HTN3**. **HTN2** is similar in EC_50_ and maximal activity to **PBN**. However, the EC_50s_ of these **HTNs** were higher than their corresponding **HBNs 4-6** [46] (Table 1), which indicates that, having a similar maximum activity, **HTNs** showed less neuroprotective capacity than their structurally corresponding **HBNs 4-6 [46]** and **NAC [46]**. Therefore, we conclude that the introduction of an additional nitrone group seemed not to provide an additional benefit to the neuroprotective activity observed for **HBNs 4-6**, bearing two nitrone groups.

#### 2.2.2. Neuroprotection Analysis in an Oxygen Glucose Deprivation Model

**Figure 3:**
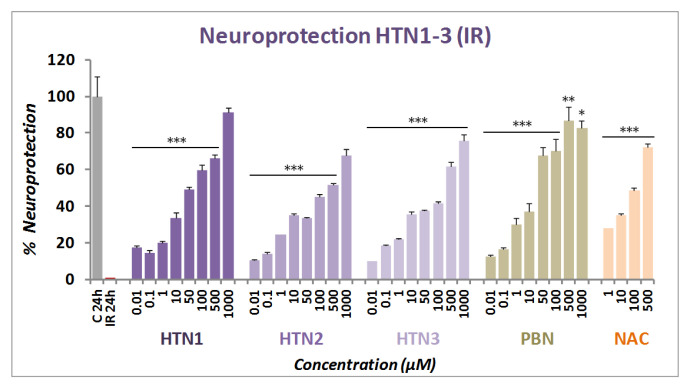
Neuroprotective effect of HTNs 1-3 on neuroblastoma cell viability after oxygen glucose deprivation (4 h) and ischemic reperfusion (24 h) (IR). Bars show % neuroprotection at indicated concentrations, considering 100% neuroprotection the difference in viability between Control (C_24h_) and IR alone. Values represent the mean ± SEM after three experiments, each one in triplicate. The statistics compare the effect of each HTN concentration with 100% neuroprotection, without IR (C 24h) at * *p* < 0.05, ** *p* < 0.01, and *** *p* < 0.001 (ANOVA one-way, followed by Holm−Sidak analysis as test post hoc).

The neuroprotective effect of **HTNs 1-3** was next evaluated in an in vitro oxygen glucose deprivation (OGD) model, followed by ischemic reperfusion (IR) by using XTT cell viability test, a colorimetric assay that detects the cellular metabolic activities, at concentrations from 0.01 to 1000 μM, after IR. After OGD (I) (4 h), 50–80% cell death was observed, showing a small cell recovery after 24 h IR of 38–61% (49.29 ± 3.26; mean ± SEM; *n* = 16). As shown in Figure 3, all **HTNs 1**-**3** were able to partially or totally reverse cell death induced by IR, in a concentration dependent manner.

Based on these results, next we carried out the analyses of concentration–response curves for all the **HTNs**, **PBN**, and **NAC**, in the 0.00001 and 1 mM range, taking into account the potential neurotoxic effect at high concentrations, as measured in the basal neurotoxicity section (see below). As a result, we determined the EC_50_ and the highest neuroprotective activities for **HTNs 1**-**3**, and **PBN**. As shown in Table 2, nitrones **HTNs 1**-**3** were less potent neuroprotective agents than **PBN**, as they showed significantly higher EC_50_ values_,_ although they exhibited higher neuroprotective activity than **PBN** at higher concentrations. Thus, the order of neuroprotective power (from lowest to highest EC_50_) was **PBN** ≥ **HTN2** ≥ **HTN3** ≥ **HTN1** (Table 2). In this case, there was only a small significant difference between the EC_50_ values of **HTN2** and **HTN1**, but there were no significant differences between the % of maximal neuroprotective activities among the **HTNs 1**-**3**. However, like in the neuroprotection against O/R, the EC_50s_ values of these **HTNs** were higher than their corresponding **HBNs 4**-**6** and **NAC,** having a similar maximal activity [46] (Table 2), which indicates that, in this case too, the introduction of a third nitrone group, does not provide a better neuroprotective capacity with respect to their structurally similar **HBNs**. Thus, from the structure activity relationship (SAR) point of view, note that the incorporation of a new nitrone group in **HTNs 1-3** with respect to **HBN4-6 [46]**, significantly decreased their neuroprotective capacity.

#### 2.2.3. Effect of HTNs on Necrotic and Apoptotic Cell Death Induced by Induced by Oxygen Glucose Deprivation

During an ischemic stroke, in the brain area affected there is massive cell death due to necrosis, and as a consequence, the plasma membrane is broken or significantly permeabilized. Under these circumstances, lactate dehydrogenase (LDH), a soluble cytosolic enzyme, easily crosses the damaged membrane, and by measuring its extracellular activity, and comparing it with its intracellular activity, it is possible to determine the extent of the cell necrosis that took place in the OGD experiment.

**Figure 4:**
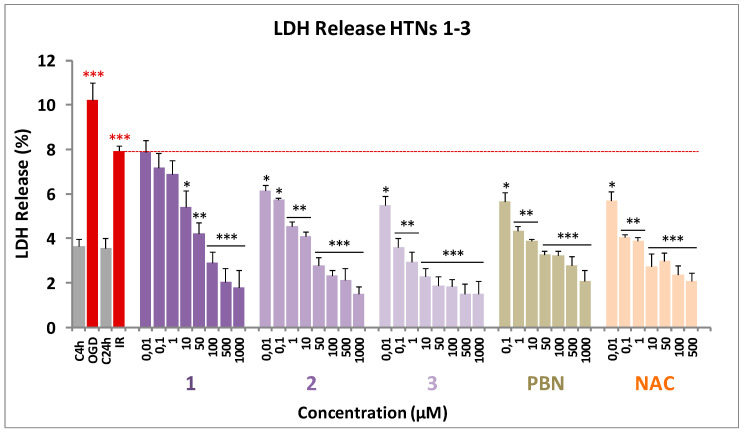
Effect of HTNs 1-3 on the lactate dehydrogenase release in SH-SY5Y cells after ischemia reperfusion. Bars show % LDH release after OGD (4 h) and IR (24 h), without treatment (IR 24 h) or treated with **HTNs 1-3**, **PBN**, and **NAC**, at the indicated concentrations. Values are the mean ± SEM of three experiments, each one performed in triplicate, and compared to the effect of I and IR on respective controls (red ***) or the effect of the different compounds after IR (24 h) with IR (24 h) alone (red dotted line) in the absence of these compounds (black ***). Data were statistically analyzed by one-way ANOVA, followed by Holm–Sidak as test post hoc. * *p* < 0.05, ** *p* < 0.01, and *** *p* < 0.001.

As shown in Figure 4, from the values obtained from the measurement of the LDH release after OGD for 4 h followed by 24 h IR, on neuroblastoma cells, by adding the **HTNs**, **PBN**, and **NAC**, at 1–1000 µM concentrations, we concluded that the more efficient was **HTN3**, followed by **HTN2** and **HTN1** (Figure 4).

These data were confirmed by the calculation of the IC_50_ and the maximal activities (Table 3). In this case, **HTNs 1-3** showed similar maximal activity; so, their efficiency was compared using the IC_50_ values.

According to the results in Table 3 **HTN3** exhibits higher antinecrotic power (lower IC_50_) than **HTN2**, followed by **HTN1**, which had the lowest antinecrotic power of the tested **HTNs**. Comparing with the standards, **HTN3** is significantly 10- and 6-fold more potent than **PBN** and **NAC**, respectively.

Next, and in order to evaluate the extent of cell death by apoptosis, we determined the caspase-3 activity, by using DEVD-AMC as substrate, that when hydrolyzed affords fluorescent AMC. Therefore, after OGD (4 h), and adding **HTNs 1**-**3**, **PBN**, and **NAC**, at 1–250 μM concentration doses, followed by IR (24 h), the cells were lysated, DEVD-AMC was added, and the fluorescence measured.

**Figure 5:**
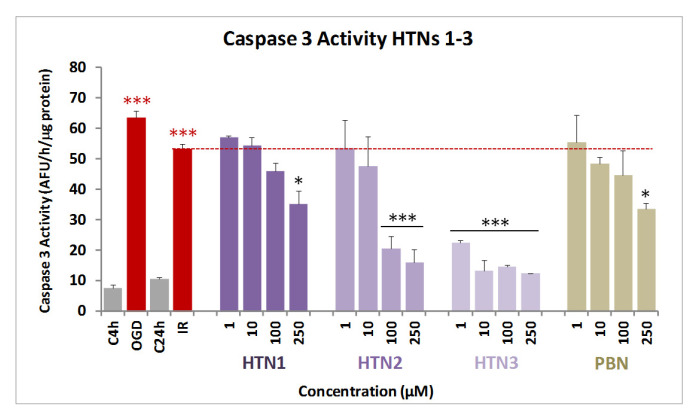
Effect of HTNs 1-3 on caspase-3 activity in SH-SY5Y neuroblastoma cells after ischemia reperfusion. Bars show % LDH release after OGD (4 h) and IR (24 h), without treatment (IR alone) or treated with **HTNs 1-3**, **PBN**, and **NAC**, at the indicated concentrations. Values are the mean ± SEM of three experiments, each one performed in triplicate, and compared to the effect of I and IR on respective controls (red ***) or the effect of the different compounds after IR (24 h) with IR alone (red dotted line), in the absence of these compounds (black ***). Data were statistically analyzed by one-way ANOVA, followed by Holm–Sidak as test post hoc. * *p* < 0.05, ** *p* < 0.01, and *** *p* < 0.001. UAF = arbitrary fluorescent units.

As shown from the values in Figure 5, and regarding antiapoptotic effects, compound **HTN3** was undoubtedly the most potent of the three **HTNs**, even more active than its homologous **HBN6** [46]. Thus, we can conclude that the *N*-Bn group provides antiapoptotic activity to these nitrones. Finally, as it was the case with their antinecrotic capacity, **HTN3** was significantly higher than that of **PBN**, while the antiapoptotic activity of **HTN1** was similar, and not significantly different, than that of the reference nitrone.

#### 2.2.4. Basal Neurotoxicity of HTNs 1-3

**Figure 6:**
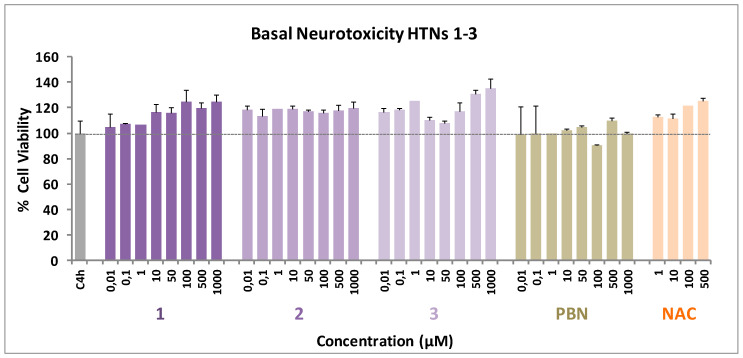
Effect of HTNs 1-3, PBN, and NAC on human neuroblastoma SH-SY5Y cell viability under basal conditions. Bars represent % of cell viability in the presence of the ligands at the indicated concentrations. Cell viability for the untreated cells (C4h) was assigned 100% (100 ± 5.52%). Values are the mean ± SEM of five experiments, each one in triplicate. No statistically significant differences were observed between the control (black dotted line) and the different nitrones tested at the concentrations indicated (one-way ANOVA test).

Based on the results described above, the analysis of the possible neurotoxicity of the **HTNs 1**-**3** was mandatory. Thus, an experiment was carried out by measuring the cell viability with XTT, but without adding any toxic insult. As shown in Figure 6, **HTN1**-**3**, as well as **PBN** and **NAC**, did not show any neurotoxic effect at basal level.

#### 2.2.5. Antioxidant Capacity of HTNs 1-3. Production and Scavenging of Radical Superoxide Radical in Human Neuroblastoma SH-SY5Y Cells

The results shown in the previous sections prompted us to investigate whether the observed neuroprotection due to **HTNs 1**-**3** was a consequence of their capacity to act as antioxidants, and ROS scavengers, particularly of superoxide anion-radical (O_2_^−λ^), whose detection can be carried out by using dihydroethidium (DHE), after OGD (3 h) and IR (3 h), with, or without **HTNs 1**-**3**, including **PBN** as a reference.

**Figure 7:**
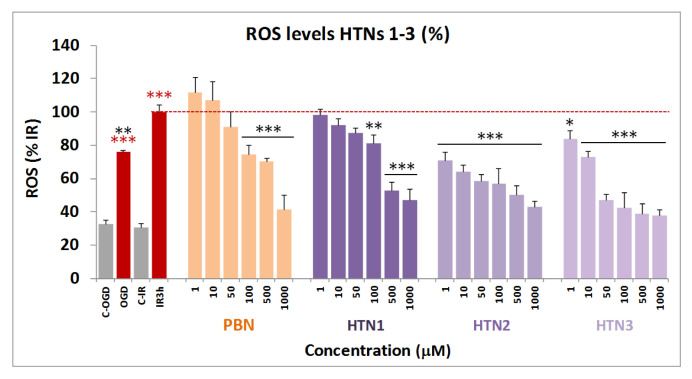
Inhibitory effects of HTNs 1-3, and PBN on ROS (superoxide) production in SHSY5Y human neuroblastoma cell cultures exposed to oxygen glucose deprivation (4 h) and ischemic reperfusion (3 h). Bars shows % ROS formed after OGD and IR alone (IR 3 h) or after treatment with HTNs 1-3 and PBN, at the indicated concentration concentrations. Values are then mean ± SEM of three experiments, each one performed in triplicate. Values for ROS in IR were calculated as 1.98 ± 0.25 UAF/min/150,000 cells. The statistics compare the effect of IR against the corresponding control (red ***) or the effect of the different compounds respect to IR alone (red dotted line), (black ***) at * *p* < 0.05, ** *p* < 0.01, *** *p* < 0.001 (one-way ANOVA followed by Holm–Sidak analysis post hoc).

As shown in Figure 7, ROS level production after IR was higher than ROS production after OGD alone. As expected, **PBN** was a middle ROS trapping agent compared to the tested nitrones. Thus, in general, the **HTNs 1**-**3** showed an excellent antioxidant profile, the most potent being **HTNs 2** and **3**, with an antioxidant profile similar to their counterparts **HBN5** and **6,** [46] greatly exceeding the antioxidant power of **PBN**.

The analyses of concentration–response curves and calculations of EC_50_ and the highest antioxidant activities for **HTNs 1–3, PBN**, and **NAC** are shown in Table 4. The EC_50_ values, from the lowest to the highest, follows the order: **NAC ≤ HTN2 ≤ HTN3 < PBN < HTN1.** As the highest antioxidant activity (maximal activities) was lower for **HTN1** and **PBN** and higher for **HTNs 2**, **3**, and **NAC,** we conclude that, regarding the antioxidant capacity against IR-induced superoxide production, **HTN2** exhibit the best antioxidant properties, which was very similar to the antioxidant capacity of its **HBNs 5** and **6** counterparts [46], as well as that of **NAC** (Table 4).

In summary, and from the SAR point of view, once again **HTN2** and **HTN3**, bearing *N-tert*-Bu and *N*-Bn, substituents, respectively, were confirmed to be the most potent **HTNs**, with antioxidant capacity similar to their analogous **HBNs 5** and **6**, and to **NAC**, and better than that of **PBN**.

### 2.3. Antioxidant Tests

Based on the in vitro neuroprotection results, we carried out the antioxidant power analysis of **HTNs 1-3**, on diverse antioxidant tests [53], using NDGA and Trolox as standards for comparative purposes. As shown in Table 5, **HTN3** was able to inhibit 92% LP, in the same range as Trolox (88%), in the same experiment, as well as LOX (70 μM) and ABTS^+.^ (10%), albeit to a poorer extent than NDGA (0.45 μM), and Trolox (91%), respectively. **HTN2** also has a good antioxidant profile, although weaker than **HTN3**, as it was only able to inhibit 55% LP, as well as LOX (26%), but not (ABTS^+.^). However, **HTN3** and **HTN2** were the most potent hydroxyl radical scavengers (83% and 81%, respectively), very similar to **HBNs 5** and **6** [46], respectively, and in the same range as Trolox (83%). Finally, among **HTNs** and compared to **PBN**, **HBNs 5** and **6**, **HTN3** and **HTN2** showed the most potent balanced antioxidant capacity.

## 3. Materials and Methods

### 3.1. Chemistry

#### 3.1.1. General Methods

Compound purification was performed by column chromatography with Merck Silica Gel (40–63 µm) or by flash chromatography and the adequate eluent for each case. Reaction course was monitored by thin layer chromatography (t.l.c.), revealing with UV light (λ = 254 nm) and ethanolic solution of vanillin or ninhydrin. Melting points were determined using a Reichert Thermo Galen Kofler block and were uncorrected. Samples were dissolved in CDCl_3_ or DMSO-*d_6_* using TMS as internal standard for ^1^H NMR spectra. In ^13^C NMR spectra, CDCl_3_ central signal (77.0 ppm) and DMSO-*d* (39.5 ppm) were used as references. ^1^H-NMR and ^13^C-NMR spectra were obtained in Bruker Avance 300 (300 MHz) and Bruker Avance 400 III HD (400 Hz) spectrometers. Chemical shifts (δ) are given in ppm. Coupling constants (*J*) are given in Hz. Signal multiplicity is abbreviated as singlet (s), doublet (d), triplet (t), quartet (c), doublet of doublets (dd), triplet of doublets (td), or multiplet (m). IR spectra were recorded on a Perkin-Elmer Spectrum One B spectrometer. Units are cm^−1^. Low resolution mass spectra were recorded on an Agilent HP 1100 LC/MS Spectrometer, whereas High Resolution mass spectrometry (Exact Mass) was performed in an AGILENT 6520 Accurate-Mass QTOF LC/MS Spectrometer. Elemental analysis was performed in an Elementary Chemical Analyzer LECO CHNS-932.

#### 3.1.2. General Method for the Synthesis of HTNs

To a suspension of benzene-1,3,5-tricarbaldehyde (1 mmol) in dry THF (20 mL), anhydrous NaHCO_3_ (3 equiv), Na_2_SO_4_ (4 equiv) and the corresponding *N*-alkylhydroxylamine hydrochloride (3 equiv) were added. The mixture was irradiated at 90 °C, and 15 bar, for the time indicated in each case. Then, the mixture was cooled, the solvent removed, and the crude purified by column chromatography.

#### 3.1.3. (1. Z,1′Z,1″Z)-1,1′,1″-(Benzene-1,3,5-triyl)tris(N-methylmethanimine oxide) (HTN1)

Following the general method, the reaction of benzene-1,3,5-tricarbaldehyde (113,4 mg, 0,7 mmol) with NaHCO_3_ (229,3 mg, 2,73 mmol), Na_2_SO_4_ (397,6 mg, 2,73 mmol), and *N*-methylhydroxylamine hydrochloride (228,1 mg, 2,73 mmol), in THF (20 mL), for 6 h, after work-up and purification by column chromatography eluting with MeOH:CH_2_Cl_2_ 8%, gave **HTN1** (123,8 mg, 71%): mp > 220 °C; IR (KBr) ν 3422, 1598, 1412, 1187, 1158 cm^−1^; ^1^H NMR (400 MHz, DMSO-d_6_) δ 9.11 (s, 3 H, H-7, H-9, H-11), 7.96 (s, 3 H, H-2, H-4, H-6), 3.81 (s, 9 H, CH_3_); ^13^C NMR (101 MHz, DMSO-d_6_) δ 133.9 (3 C, C-2, C-4, C-6), 131.7 (3 C, C-1, C-3, C-5), 128.2 (3 C, C-7, C-9, C-11), 54.6 (3 C, CH_3_); MS (ES) m/z (%): 249 [M+1]^+^, 271 [M+Na]^+^. Anal. Calcd for C_12_H_15_N_3_O_3_.2/3H_2_O: C, 55.16; H, 6.30; N, 16.08. Found: C, 55.12; H, 6.12; N, 15.89.

#### 3.1.4. (1. Z,1′Z,1″Z)-1,1′,1″-(Benzene-1,3,5-triyl)tris(N-tert-butylmethanimine oxide) (HTN2)

Following the general method, the reaction of benzene-1,3,5-tricarbaldehyde (81 mg, 0.5 mmol) with NaHCO_3_ (163.8 mg, 1.95 mmol), Na_2_SO_4_ (284 mg, 2 mmol), and *N*-*t*-butylhydroxylamine hydrochloride (244.9 mg, 1.95 mmol), in THF (20 mL), for 15.5 h, after work-up and purification by column chromatography eluting with AcOEt, gave **HTN2** (123.8 mg, 59%): mp > 220 °C; IR (KBr) ν 3434, 2975, 1562, 1361, 1172 cm^−1^; ^1^H NMR (400 MHz, DMSO-*d*_6_) δ 9.33 (s, 3 H, H-7, H-9, H-11), 7.86 (s, 3 H, H-2, H-4, H-6), 3.33 (s, 27 H, C(CH_3_)_3_]; ^13^C NMR (101 MHz, DMSO-*d*_6_) δ 131.8 (3 C, C-1, C-3, C-5), 129.5 (3 C, C-7, C-9, C-11), 128.8 (3 C, C-2, C-4, C-6), 71.1 (3 C, C-8, C-10, C-12), 28.3 [9 C, C(CH_3_)_3_]; MS (ES) *m/z* (%): 376 [M+1]^+^, 398 [M+Na]^+^. Anal. Calcd for C_21_H_33_N_3_O_3_: C, 67.17; H, 8.86; N, 11.19. Found: C, 66.94; H, 8.87; N, 11.07.

#### 3.1.5. (1. Z,1′Z,1″Z)-1,1′,1″-(Benzene-1,3,5-triyl)tris(N-benzylmethanimine oxide) (HTN3)

Following the general method, the reaction of benzene-1,3,5-tricarbaldehyde (81 mg, 0.5 mmol) with NaHCO_3_ (163.8 mg, 1.95 mmol), Na_2_SO_4_ (284 mg, 2 mmol), and *N*-benzylhydroxylamine hydrochloride (310.1 mg, 1.95 mmol), in THF (20 mL), for 6 h, after work-up and purification by column chromatography eluting with AcOEt, gave **HTN3** (203.2 mg, 85%): mp 165−7 °C; IR (KBr) ν 3429, 1580, 1580, 1496, 1457, 1176 cm^−1^; ^1^H NMR (400 MHz, DMSO-d6) δ 9.13 (s, 3 H, H-7, H-10, H-13), 8.18 (s, 3 H, H-2, H-4, H-6), 7.54–7.29 (m, 15 H, C_6_H_5_), 5.09 (s, 6 H, H-7, H-11, H-14); ^13^C NMR (101 MHz, DMSO-d_6_) δ 135.1 (3 C, C_6_H_5_), 133.2 (3 C, C-2, C-4, C-6), 131.6 (3 C, C-1, C-3, C-5), 129.5 (6 C, C_6_H_5_), 128.9 (6 C, C_6_H_5_), 128.8 (3 C, C_6_H_5_), 128.6 (3 C, C-7, C-10, C-13), 70.6 (3 C, C-7, C-11, C-14); MS (ES) *m/z* (%): 478 [M+1]^+^, 500 [M+Na]^+^. Anal. Calcd for C_30_H_27_N_3_O_3_.1/5H_2_O: C, 74.89; H, 5.74; N, 8.73. Found: C, 74.87; H, 5.81; N, 8.76.

### 3.2. Neuroprotection Assessment Assays

#### 3.2.1. Neuroblastoma Cell Cultures

The human neuroblastomas cell line SH-SY5Y were cultured in Dulbecco’s: Ham’s F12, 1:1 [vol/vol] containing 3.15 mg/mL glucose, 2.5 mM Glutamax and 0.5 mM sodium pyruvate DMEM/F-12, GlutaMAX™; GIBCO, Life Technologies, Madrid (Spain), 1% Antibiotique-Antimitotic (Gibco; Life Technologies, Madrid, Spain) (containing 100 ui/mL penicillin, 100 mg/mL of streptomycin and 0.25 mg of amphotericine B), 1% gentamicina 15 mg/mL (Sigma-Aldrich, Madrid, España) and 10% Foetal Calf Serum (FCS) (Gibco; Life Technologies, Madrid, Spain) as described [46,49]. Cultures were seeded into flasks containing supplemented medium and maintained at 37 °C in a humidified atmosphere of 5% CO_2_ and 95% air. Culture media were changed every 2 d. Cells were subcultured after partial digestion with 0.25% trypsin-EDTA. For assays, SHSY5Y cells were subcultured in 96- or 48-well plates at a seeding density of 0.50–1 or 2–2.5 × 10^5^ or cells per well, respectively. When the SHSY5Y cells reached 80% confluence, the medium was replaced with fresh medium containing 0.01–1000 μM compound concentrations or PBS in the controls, as indicated in each assay.

#### 3.2.2. Neuroblastoma Cell Cultures Exposure to Oxygen Glucose Deprivation

Neuroblastoma cell cultures were exposed to OGD so as to induce cellular damage (experimental ischemia) as described [46]. Cultured cells were washed and placed in glucose-free Dulbecco’s medium (bubbled with 95% N_2_/5% CO_2_ for 30 min) and maintained in an anaerobic chamber containing a gas mixture of 95% N2/5% CO_2_ and humidified at 37 °C at a constant pressure of 0.15 bar. Cells were exposed to OGD for a period of 4 h (OGD 4 h), as indicated. At the end of the OGD period, culture medium was replaced with oxygenated serum-free medium, and cells were placed and maintained in the normoxic incubator for 24 h to recovery (R24h). In the neuroprotection experiments, **HTNs 1**-**3** and **PBN** (0.01 μM−1 mM) were added at the beginning of recovery period (see below). Control cultures in Dulbecco’s medium containing glucose were kept in the normoxic incubator for the same period of time as the OGD (C4h), and then culture medium was replaced with fresh medium and cells were returned to the normoxic incubator until the end of recovery period (C24h). Control experiments included the same amounts of vehicle (final concentration < 0.01% dimethyl sulfoxide). The experimental procedures were blindly performed, assigning a random order to each assayed nitrone. Nitrones were analyzed independently 3–5 times with different batches of cultures, and each experiment was run in triplicate.

#### 3.2.3. Assessment of Cell Viability

Measurements of cell viability in human SHSY5Y neuroblastoma cells were carried out into 96- well culture plates as described [46,50]. Briefly, control and treated SH-SY5Y neuroblastoma cells (about 0.75–1 × 105 cells/well) were incubated with the XTT solution (Cell Proliferation Kit II (XTT), Sigma, Aldrich, Madrid) at 0.3 mg/mL final concentration for 2 h in a humidified incubator at 37 °C with 5% CO_2_ and 95% air (*v*/*v*) and the soluble orange formazan dye formed was spectrophotometrically quantified, using a Biotek Power-Wave XS spectrophotometer microplate-reader at 450 nm (reference 650 nm). All XTT assays were performed in triplicate in cells of at least three different cell batches. Control cells treated with DMEM alone were regarded as 100% viability. Controls containing different DMSO concentrations (0.001–1% DMSO) were performed in all assays.

#### 3.2.4. Measurement of LDH Activity

For these assays, cultured neuroblastoma cells grown in 96-well culture dishes at a density of 1.5 × 105 cells/well were used. LDH activity was measured as the rate of decrease of the absorbance at 340 nm, resulting from the oxidation of NADH to NAD+ as described [46,51]. Data are given as the percentage of LDH release with respect to the total LDH content (LDH in the culture medium and LDH inside the cells).

#### 3.2.5. Analysis of Caspase-3 Activity

For these assays, cultured neuroblastoma cells grown in 48-well culture dishes, at a density of 2.5 × 10^5^ cells/well, were used. After OGD treatment, cells were treated with different nitrones or indicated positive controls at 1−500 μM concentrations and subjected to 24 h IR. Attached cells were lysed at 4 °C in a lysis medium containing 5 mM Tris/HCl (pH 8.0), 20 mM ethylenediaminetetraacetic acid, and 0.5% Triton X-100 and centrifuged at 13,000g for 10 min. The activity of caspase- 3 was measured using the fluorogenic substrate peptide DEVD-amc (66081; BD Biosciences PharMingen), as described [46,51]. Proteins were measured by the Bradford assay. Results were expressed as arbitrary fluorescence units [(AFU)/μg protein/h].

#### 3.2.6. Measurement of Reactive Oxygen Species Formation

SHSY5Y human neuroblastoma cells (2 × 10^5^ cells/well) were exposed to OGD for a period of 4 h (OGD4h). At the end of the OGD period, the culture medium was replaced with oxygenated Dulbecco’s modified Eagle’s medium containing glucose and 10% fetal calf serum. Cells were treated in the absence (controls) or presence of indicated concentrations of nitrones or different known neuroprotective agents and maintained at 37 °C in a normoxic incubator for 3 h for recovery. At the end of this period, 20 μM DHE (HEt; Molecular Probes) was added and fluorescence was recorded every 15−30 s during a 15 min period, using an excitation filter of 535 nm and an emission filter of 635 nm in a spectrofluorimeter (Bio-Tek FL 600) as previously described [46,52]. Linear regression of fluorescence data [expressed as arbitrary fluorescence units (AFU)] was calculated for each condition, and the slopes (a) of the best fitting lines (y = ax) were considered as an index of O_2_^•−^ production [52].

#### 3.2.7. Statistical Analysis

Data were expressed as mean ± SEM of results obtained from at least three independent experiments from different cultures, each of which was performed in triplicate. Statistical comparisons between the different experimental conditions were performed using one-way analysis of variance (ANOVA), followed by Holm−Sidak’s post -test when the analysis of variance was significant. A *p* value < 0.05 was considered statistically significant. Fit curves for EC_50_ determinations were performed according to the program of SigmaPlot v.11 (Systat Software Inc., Germany), 2012).

### 3.3. Antioxidant Activity Tests of HTNs 1-3, PBN, Trolox, and NDGA in Vitro

#### 3.3.1. Estimation of Lipophilicity as Clog P

Bioloom of Biobyte Corp was used for the theoretical calculation of lipophilicity as Clog *p* values (BioByte Home Page. Available online: http://www.biobyte.com).

#### 3.3.2. General Methods

Nordihydroguaiaretic acid (NDGA), Trolox, 2,2′-azobis(2-amidinopropane) dihydrochloride (AAPH), Soybean LOX linoleic acid sodium salt were purchased from the Aldrich Chemical Co. Milwaukee, WI, (USA). Phosphate buffer (0.1 M and pH 7.4) was prepared mixing an aqueous KH_2_PO_4_ solution (50 mL, 0.2 M), and an aqueous of NaOH solution (78 mL, 0.1 M); the pH (7.4) was adjusted by adding a solution of KH_2_PO_4_ or NaOH). For the in vitro tests a Lambda 20 (Perkin–Elmer-PharmaSpec 1700) UV–Vis double beam spectrophotometer was used.

#### 3.3.3. Inhibition of Linoleic Acid Peroxidation

For initiating the free radical, 2,2′-azobis(2-amidinopropane) dihydrochloride (AAPH) was used. The final solution in the UV cuvette consisted of 10 μL of the 16 mM linoleate sodium dispersion, 0.93 mL of 0.05 M phosphate buffer, pH 7.4, thermostated at 37 °C. A total of 50 μL of 40 mM AAPH solution was added as a free radical initiator at 37 °C under air and 10 μL of the tested compounds. The oxidation of linoleic acid sodium salt results a conjugated diene hydroperoxide. The reaction was monitored at 234 nm.

#### 3.3.4. Soybean Lipoxygenase Inhibition Study

DMSO solution of the tested compound (stock solutions 10 mM) was incubated with sodium linoleate (0.1 mM) and 0.2 mL of soybean lipoxygenase solution (1/9 × 10^−4^ *w/v* in saline) at room temperature. The test followed our previous published methods [53]. The conversion of sodium linoleate to 13-hydroperoxylinoleic acid was recorded at 234 nm and compared with the standard inhibitor NDGA (ΙC_50_ = 0.45 μΜ/935 at 100 μΜ). Several concentrations were used to determine the IC_50_.

#### 3.3.5. ABTS^+•^—Decolorization Assay for Antioxidant Activity

The ABTS radical cation (ABTS^+•^) was generated from an ABTS stock solution in water (7 mM) which was mixed with potassium persulfate (2.45 mM) and left in the dark at room temperature for 12–16 h before use. The results were recorded after 1min of the mixing solutions at 734 nm and compared to the appropriate standard inhibitor Trolox [53].

#### 3.3.6. Hydroxyl Radical Scavenging Activity

The hydroxyl radicals were produced by the Fe^3+^/ascorbic acid system. EDTA (0.1 mM), Fe^3+^ (167 μM), DMSO (33 mM) in phosphate buffer (50 mM, pH 7.4), the tested compounds (0.1 mM), and ascorbic acid (10 mM) were mixed in test tubes. The solutions were incubated at 37 °C for 30 min. The reaction was stopped by CCl_3_COOH (17% *w*/*v*) and the % scavenging activity of the tested compounds for hydroxyl radicals was given.

## 4. Conclusions

In this work we described the design, synthesis, neuroprotection, and antioxidant capacity of **HTNs 1**–**3** derived from **PBN**, comparing them with **HBN6** (Figure 1), our most recently discovered potent neuroprotective homo-bis-nitrone [46]. The biological evaluation of these **HTNs** included neuroprotection against O/R, an ischemia in vitro model under OGD conditions in human neuroblastoma SH-SY5Y cells, and diverse antioxidant tests.

Our design, supported by our previous report [46], was based on the hypothesis that three nitrone motifs installed in the same scaffold should afford higher neuroprotective power than two nitrone groups. In other words, the greater the number of nitrone free radical scavenging groups, the stronger the antioxidant and neuroprotective properties.

**Figure 8:**
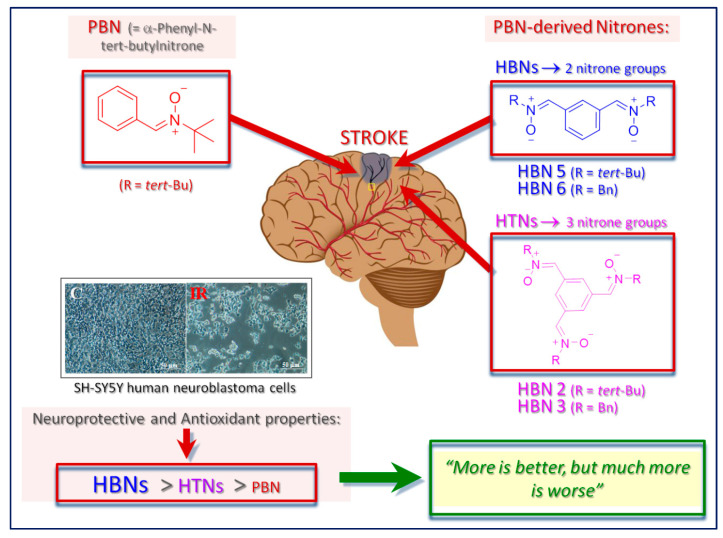
**Graphic diagram showing the most important conclusions of this work**. **HTNs 2** and **3** have a good antioxidant and neuroprotective activity against necrotic and apoptotic cell death, very similar to those of their homologous **HBN5** and **HBN6**. However, they have a worse capacity than their counterparts to reverse the cellular metabolic alterations induced by oligomycin A/rotenone and by in vitro ischemia/reperfusion in human neuroblastoma SH-SY5Y cells. Therefore, although **HTNs 2** and **3** overcome the antioxidant and neuroprotective properties of **PBN**, the addition of a third nitrone group does not improve, but rather worsens, the neuroprotective capacity observed in **HBN5** and **HBN6**. In other words, “*more is better, but much more is worse*”.

The neuroprotection of **HTNs 1-3**, measured against O/R, showed that **HTN2** was the best neuroprotective agent at a lower dose (EC_50_ = 51.63 ± 4.32 μM), but **HTN3** produced the greater neuroprotection at a higher dose (EC_50_ = 125.57 ± 6.19 μM), **HTN2** being similar in EC_50_ and maximal activity to **PBN** and less potent than any of **HBNs 4-6**. The results of neuroprotection in an in vitro oxygen glucose deprivation model showed that **HTN2** was the most powerful, EC_50_ = 87.57, 3,87 μM, at lower dose, but 50-fold higher than its analogous **HBN5**, and ≈1.7-fold less potent than **PBN**, but again **HTN3** produced the greater neuroprotection at a higher dose (EC_50_ = 124.06 ± 7.40 μM). **HTN3** has a very good antinecrotic (IC_50_ = 3.47 ± 0.57 μM), antiapoptotic, and antioxidant (EC_50_ = 6.77 ± 1.35 μM), profile, very similar to that of its analogous **HBN6**. Finally, **HTN3** was able to inhibit 92% LP, LOX (70 μM), and ABTS^+^ (10%), showing high hydroxyl radical scavenger capacity (83%) and exhibiting thus the potent balanced antioxidant capacity among the nitrones investigated here. However, the results of the present work show that in general, the **HTNs 1**-**3** do not have better properties than **HBN6**, but clearly exceed **PBN**. From the point of view of our drug design, it is clear that the introduction of a third, additional nitrone group seems not to provide an additional, accumulative benefit to the neuroprotective, and antioxidant activity previously observed for **HBN5** and **HBN6 [46]**, bearing two nitrone groups. In other words, *“more is better, but much more is worse”* (Figure 8).

Furthermore, these results confirmed what we have reported in a previous communication [54]. Thus, based on the ability of the “*N*-propargylamine” motif to impart MAO inhibition, we wondered whether the progressive incorporation of 1, 2, or 3 “*N*-propargylamine” motives in a simple core provided by the 1,3,5-triazine could afford progressive, accumulative, and higher MAO inhibition power. The observed results clearly showed again that *much more is worse*, that the incorporation of more anti-MAO pharmacophores is not a guarantee for better inhibition. On the contrary, less inhibition or no inhibition was detected, and only ligand 4,6-dichloro-*N*-(prop-2-yn-1-yl)-1,3,5-triazin-2-amine bearing a single *N*-propargylamine MAO pharmacophore motif, showed modest, but totally selective MAO-B inhibition [54].

The present result is not necessarily negative, as it was an obvious necessary development in our current program to identify new and more efficient nitrones for therapeutic purposes, and this is possibly a general trend that should be taken into account in drug discovery projects [54]. Consequently, our present endeavors in this arena are now directed to the rational and wise structural modification of **HBN5** and **HBN6** (Figure 1) to improve its neuroprotective and antioxidant properties [46].

## Figures and Tables

**Table 1:** Neuroprotective effect of **HTNs 1-3**, **PBN**, and **NAC** against neurotoxicity induced by oligomycin A/rotenone in human neuroblastoma SH-SY5Y cells.

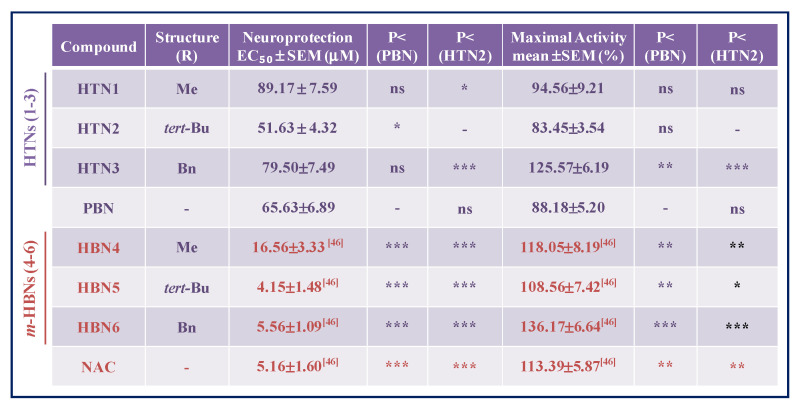

The estimation of EC_50_ (μM) and maximal activities (% neuroprotection) values were performed by a weighted nonlinear regression of minimum squares using logistic curves, as is described in the “Statistical Analysis” section of “Neuroprotection Assessment Assays”. Values are the mean ± S.E.M. Data analysis was carried out with SigmaPlot v.12., and ANOVA one-way to get the significant statistics of **HTNs** with respect to **PBN**. Differences are statistically significant when *p* ≤ 0.05. Differences are statistically significant when *p* ≤ 0.05. * *p* < 0.05, ** *p* < 0.01, and *** *p* < 0.001, ns = non-significant (ANOVA one-way, followed by Holm−Sidak analysis as test post hoc).

**Table 2:** Neuroprotective effect of **HTNs 1-3**, **PBN**, and **NAC** after oxygen glucose deprivation/ischemic reperfusion (IR) in human neuroblastoma SH-SY5Y cells.

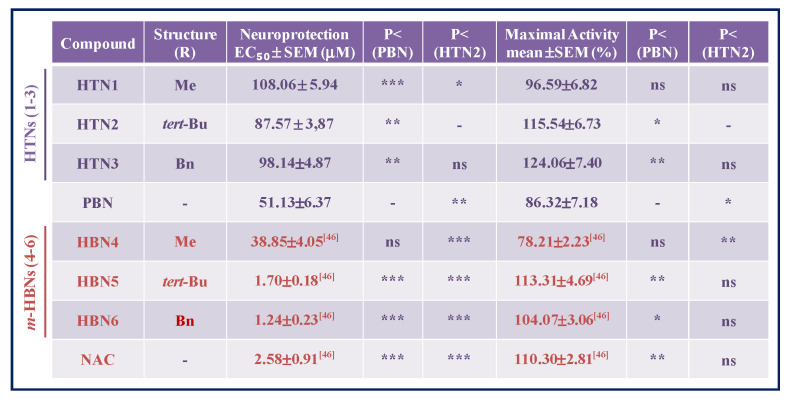

The estimation of EC_50_ (μM) and maximal activities (% neuroprotection) values were performed by a weighted nonlinear regression of minimum squares using logistic curves, as is described in the “Statistical Analysis” section of “Neuroprotection Assessment Assays”. Values are the mean ± S.E.M. Data analysis was carried out with SigmaPlot v.12., and ANOVA one-way to get the significant statistics of **HTNs** and **HBNs** with respect to **PBN** and **HTN2**. Differences are statistically significant when *p* ≤ 0.05. * *p* < 0.05, ** *p* < 0.01, and *** *p* < 0.001, ns = non-significant (ANOVA one-way, followed by Holm−Sidak analysis as test post hoc).

**Table 3:** Effect of **HTNs 1-3**, **PBN**, and **NAC** on the lactate dehydrogenase release after oxygen glucose deprivation/ischemic reperfusion in human neuroblastoma SH-SY5Y cells. EC_50_ and maximal activities.

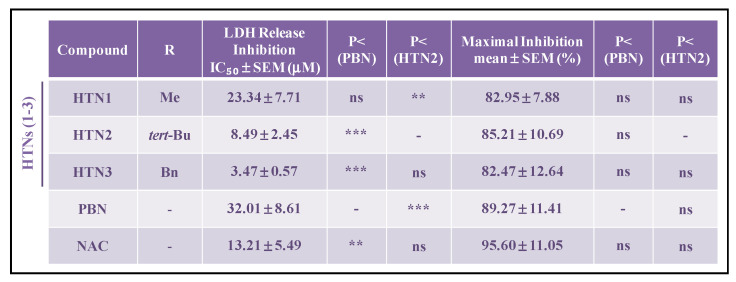

The estimation of IC_50_ (μM) and maximal inhibitory activities (%) values were performed by a weighted nonlinear regression of minimum squares using logistic curves, as is described in the “Statistical Analysis” section of “Neuroprotection Assessment Assays”. Values are the mean ± S.E.M. Data analysis was carried out with SigmaPlot v.12., and ANOVA one-way to get the significant statistics of **HTNs** with respect to **PBN**, or to **HTN2**. Differences are statistically significant when *p* < 0.05, ** *p* < 0.01, ** *p* < 0.001. ns = non-significant (ANOVA one-way, followed by Holm−Sidak analysis as test post hoc).

**Table 4:** Antioxidant effect of **HTNs 1–3**, **PBN**, and **NAC** after oxygen glucose deprivation/ischemia reperfusion in human neuroblastoma SH-SY5Y cells.

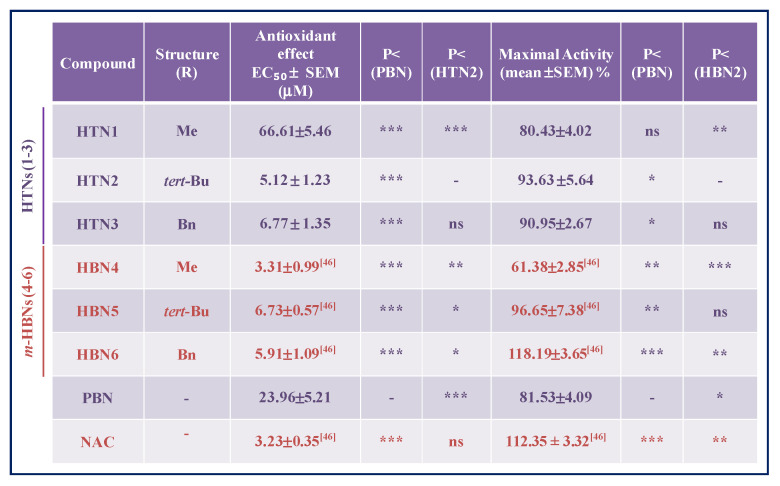

The estimation of EC_50_ (μM) and maximal activities (% antioxidant effect) values were performed by a weighted nonlinear regression of minimum squares using logistic curves, as is described in the “Statistical Analysis” section of “Neuroprotection Assessment Assays”. Values are the mean ± S.E.M. EC_50_ and maximal activities were calculated from the data obtained from three experiments, each one in triplicate. The statistics compare differences with **PBN** or **HTN2** at * *p* < 0.05, ** *p* < 0.01, and *** *p* < 0.001 and ns = non-significant (one-way ANOVA, followed by Holm–Sidak analysis as a test post hoc).

**Table 5:** Antioxidant activity of HTNs 1-3, PBN, HBNs 5, 6, Trolox, and NDGA.

Nitrones/ Standards	ClogP *	ILPO (%)	LOX Inhibition (% or IC_50_ [μM]) (±SEM)	Scavenger Activity for ^•^OH (%)	ABTS^+.^ (%)
**HTN1**	−2.94	46	100 ± 1.1 μΜ	59	No
**HTN2**	0.77	55	26 ± 0.2%	81	No
**HTN3**	2.73	92	70 ± 2.6 μΜ	83	10
**PBN**	3.02	11	23 ± 0.1%	No	5
**HBN5** [46]	4.51	55	6 ± 0.1%	67	No
**HBN6** [46]	4.96	37	29 ± 0.3%	81	No
**NDGA**			0.45 μM		
**Trolox**		88		83	91

Nitrones were tested at 100 µM. Values are means of three or four different determinations. No—no activity under the experimental conditions. Means within each column differ significantly (*p* < 0.05). nd—not determined. * Biobyte BioByte Corporation, C-QSAR database, 201 W Fourth Str., Suite 204, Claremont CA 91711-4707, USA.

## Supplementary Materials

Supplementary Materials (NMR spectra of HTNs 1-3) can be found at https://www.mdpi.com/1422-0067/21/21/7949/s1.

## Abbreviations

AAPH	2,2′-Azobis(2-amidinopropane) dihydrochloride
NAC	N-Acetyl-*L*-cysteine
BBB	Blood−brain barrier
HBNs	Homo-bis-nitrones
HTNs	Homo-tris-nitrones
IR	Ischemic reperfusion
LDH	Lactate dehydrogenase
LOX	Lipoxygenase
LP	Lipid peroxidation
MTT	3-(4,5-Dimethylthiazol-2-yl)-2,5-diphenyltetrazolium bromide
NDGA	Nordihydroguaiaretic acid
O/R	Oligomycin A/Rotenone
OGD	Oxygen Glucose Deprivation
PBN	α-Phenyl-*N*-*tert*-butylnitrone
ROS	Reactive oxygen species
