# Supplementary material for: Homo-Tris-Nitrones Derived from α-Phenyl-N-tert-butylnitrone: Synthesis, Neuroprotection and Antioxidant Properties
Source: Int J Mol Sci. 2020 Oct 26;21(21):7949. doi: 10.3390/ijms21217949 (PMC7663103; doi:10.3390/ijms21217949)

Supplementary Material

Homo-Tris-Nitrones Derived from α-Phenyl-*N*-*tert*-butylnitrone: Synthesis, Neuroprotection and Antioxidant Properties

Daniel Diez-Iriepa,[1,2,♯] Beatriz Chamorro,[3,4,♯] Marta Talaván,[3] Mourad Chioua,[1] Isabel Iriepa,[2,5] Dimitra Hadjipavlou-Litina,[6] Francisco López-Muñoz,[4,7] José Marco-Contelles[1,*] and María Jesús Oset-Gasque[3,8*]

[1] Laboratory of Medicinal Chemistry, Institute of Organic Chemistry (CSIC), Juan de la Cierva 3, 28006-Madrid, Spain.

[2] Department of Organic Chemistry and Inorganic Chemistry, Alcalá University, 28805 Alcalá de Henares, Madrid, Spain.

[3] Department of Biochemistry and Molecular Biology, Faculty of Pharmacy, Complutense University of Madrid (UCM), 28040 Madrid, Spain.

[4] Faculty of Health, Camilo José Cela University (UCJC). 28692 Villafranca del Castillo, Madrid, Spain.

[5] Institute of Chemical Research Andrés M. del Río, Alcalá University, 28805-Alcalá de Henares, Madrid, Spain.

[6] Department of Pharmaceutical Chemistry, School of Pharmacy, Faculty of Health Sciences, Aristotle University of Thessaloniki, Thessaloniki 54124, Greece

[7] Neuropsychopharmacology Unit, "Hospital 12 de Octubre" Research Institute, 28041, Madrid, Spain.

[8] Instituto Universitario de Investigación en Neuroquímica, Universidad Complutense de Madrid. Ciudad Universitaria, 28040 Madrid, Spain.

* Correspondence: mjoset@ucm.es; iqoc21@iqog.csic.es

♯ These authors have equally contributed to this work

CONTENTS

1. NMR spectra of HTNs 1-3………………………………………………………………………..S2-S4

1. NMR spectra of HTNs 1-3

^{1}H and ^{13}C NMR spectra of HTN1

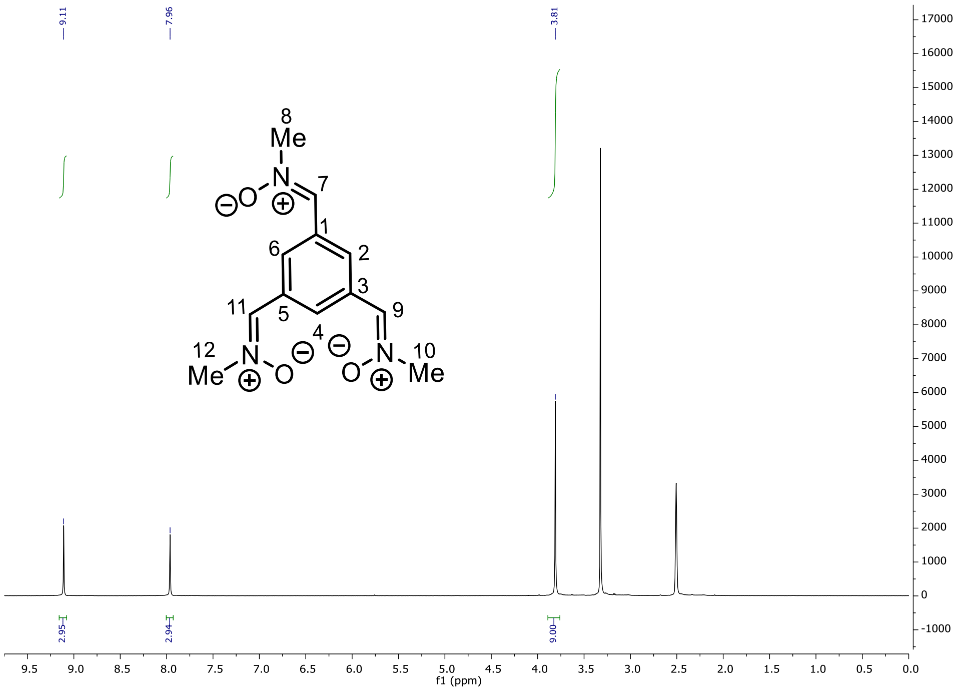

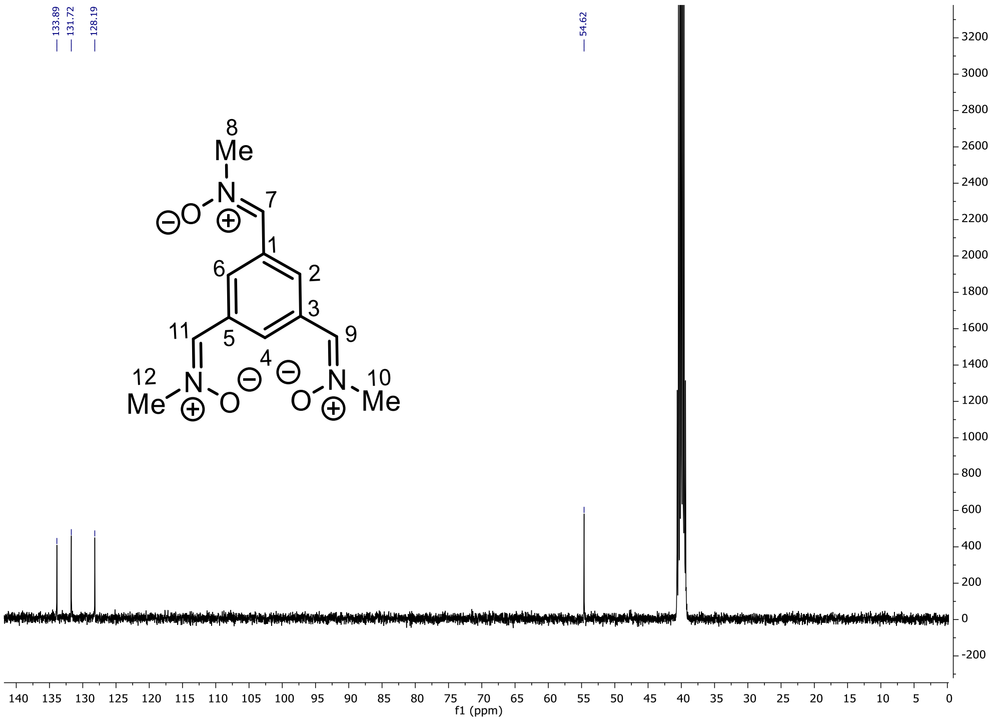

^{1}H and ^{13}C NMR spectra of HTN2

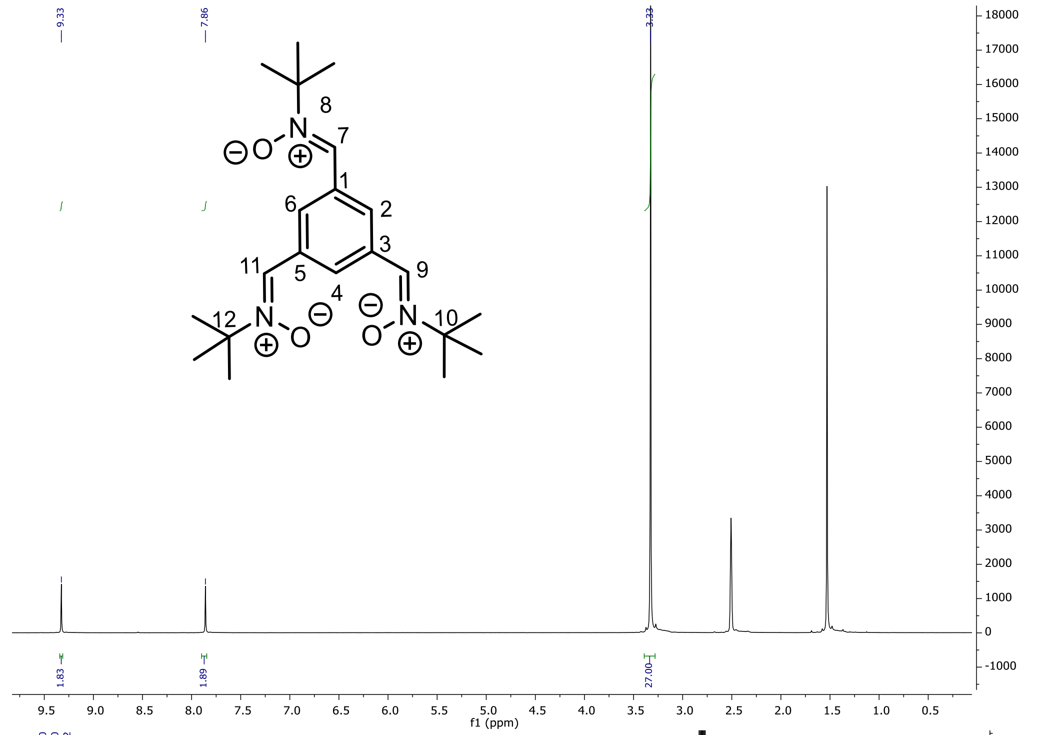

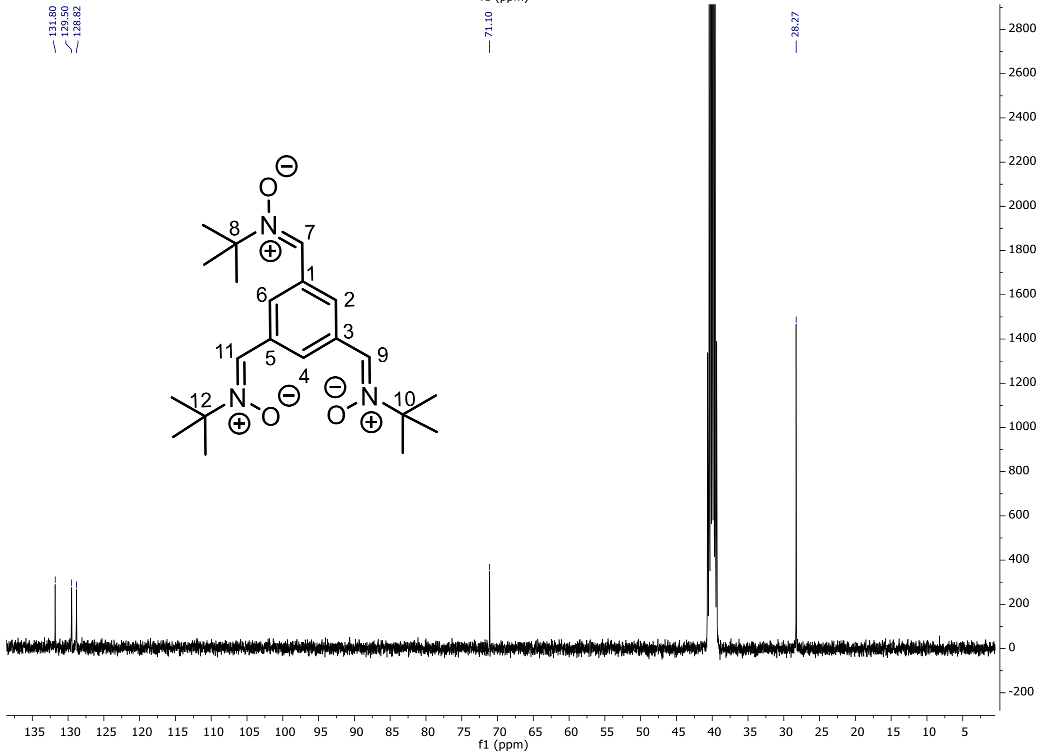

¹H and ¹³C NMR spectra of HTN3

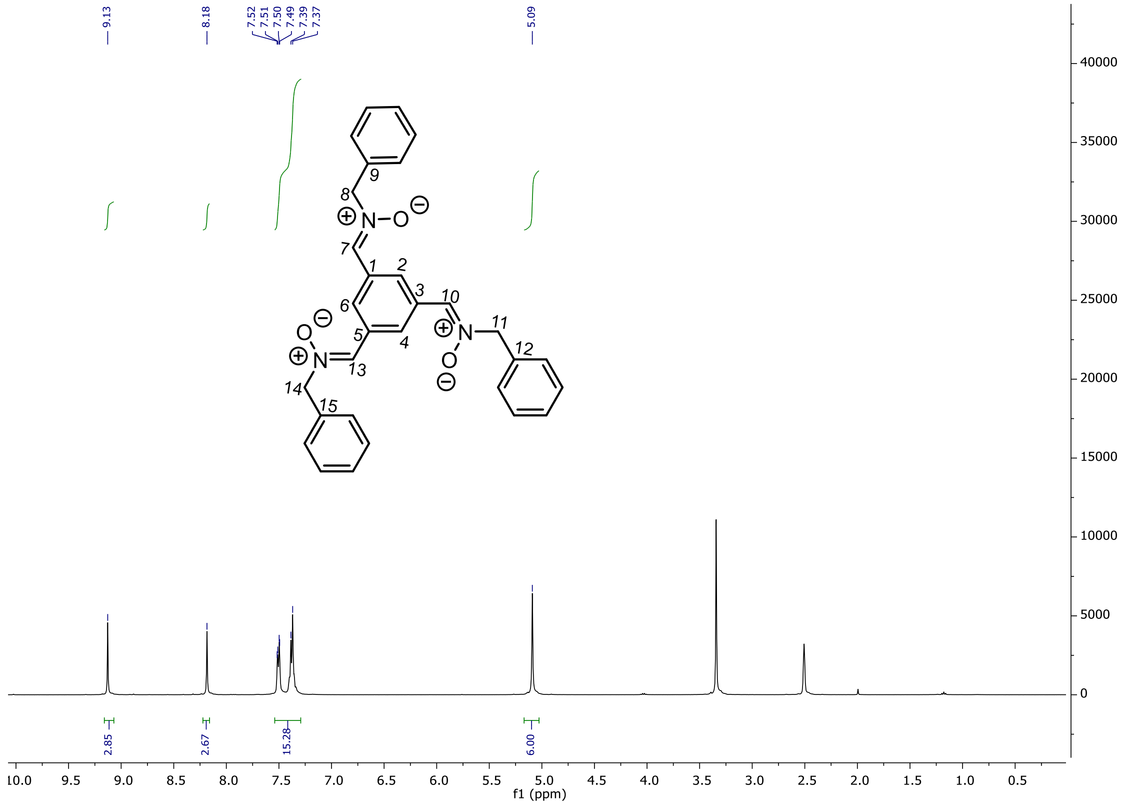

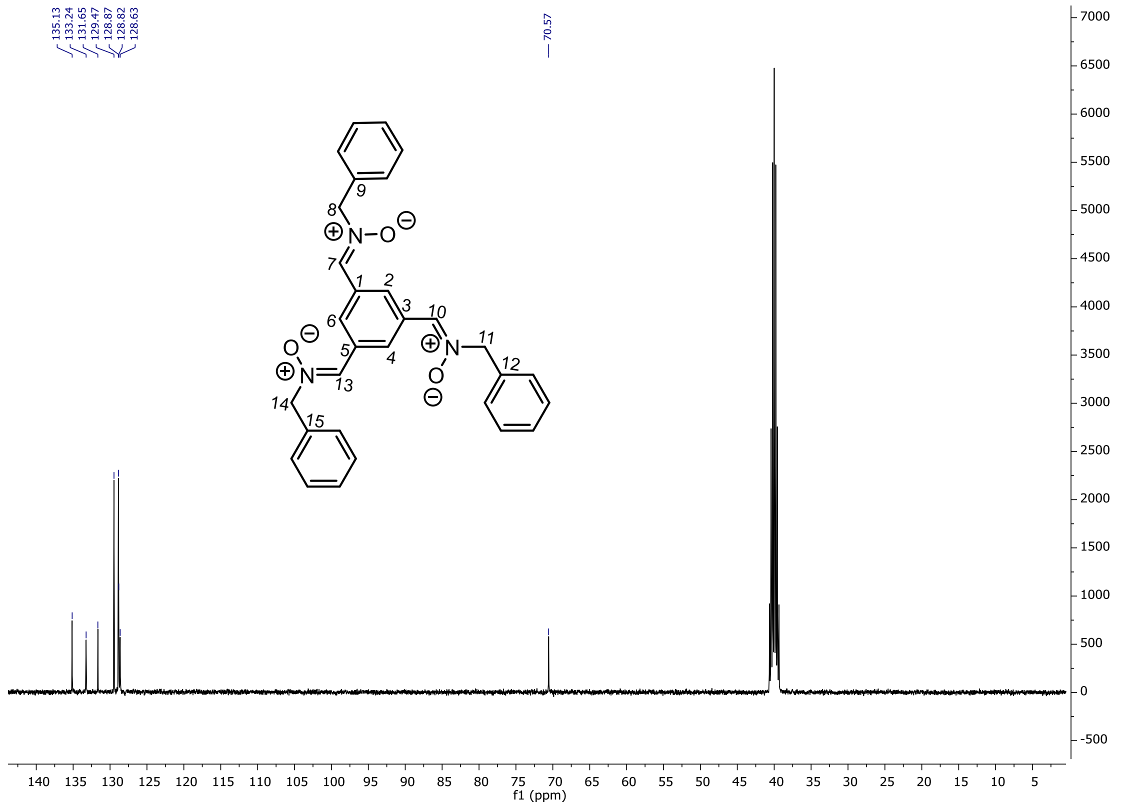

Supplement: Supplementary file 1 [file ijms-21-07949-s001.pdf]
